# Supplementary material for: Microbial short-chain fatty acids modulate CD8+ T cell responses and improve adoptive immunotherapy for cancer
Source: Nat Commun. 2021 Jul 1;12:4077. doi: 10.1038/s41467-021-24331-1 (PMC8249424; doi:10.1038/s41467-021-24331-1)
Supplement: Supplementary file 1 — Supplementary Information [file 41467_2021_24331_MOESM1_ESM.pdf]

Microbial short-chain fatty acids modulate CD8$^+$ T cell responses and improve adoptive immunotherapy for cancer

Maik Luu[1,2], Zeno Riester[2], Adrian Baldrich[2], Nicole Reichardt[3], Samantha Yuille[3], Alessandro Busetti[3], Matthias Klein[4], Anne Wempe[1], Hanna Leister[1], Hartmann Raifer[5], Felix Picard[1], Khalid Muhammad[6], Kim Ohl[7], Rossana Romero[1], Florence Fischer[1], Christian A Bauer[8], Magdalena Huber[1], Thomas M Gress[8], Matthias Lauth[9], Sophia Danhof[2], Tobias Bopp[4], Thomas Nerreter[2], Imke E Mulder[3], Ulrich Steinhoff[1], Michael Hudecek[2]*, Alexander Visekruna[1]*

[1]Institute for Medical Microbiology and Hygiene, Philipps-University Marburg, Marburg, Germany
[2]Medizinische Klinik und Poliklinik II, Universitätsklinikum Würzburg, Würzburg, Germany
[3]4DPharma Research Ltd., Aberdeen, United Kingdom
[4]Institute for Immunology, University Medical Center, Johannes Gutenberg University Mainz, Mainz, Germany
[5]Flow Cytometry Core Facility, Philipps University Marburg, Germany
[6]Department of Biology, United Arab Emirates University, Al Ain, United Arab Emirates
[7]Department of Pediatrics, RWTH Aachen University, Aachen, Germany
[8]Department of Gastroenterology, Endocrinology, Metabolism and Infectiology, University Hospital Marburg, UKGM, Philipps University Marburg, Marburg, Germany
[9]Institute of Molecular Biology and Tumor Research, Center for Tumor- and Immunobiology, Philipps-University Marburg, Marburg, Germany

Corresponding authors: Alexander Visekruna (alexander.visekruna@staff.uni-marburg.de), Michael Hudecek (Hudecek_M@ukw.de)

*These authors jointly supervised this work: Alexander Visekruna, Michael Hudecek

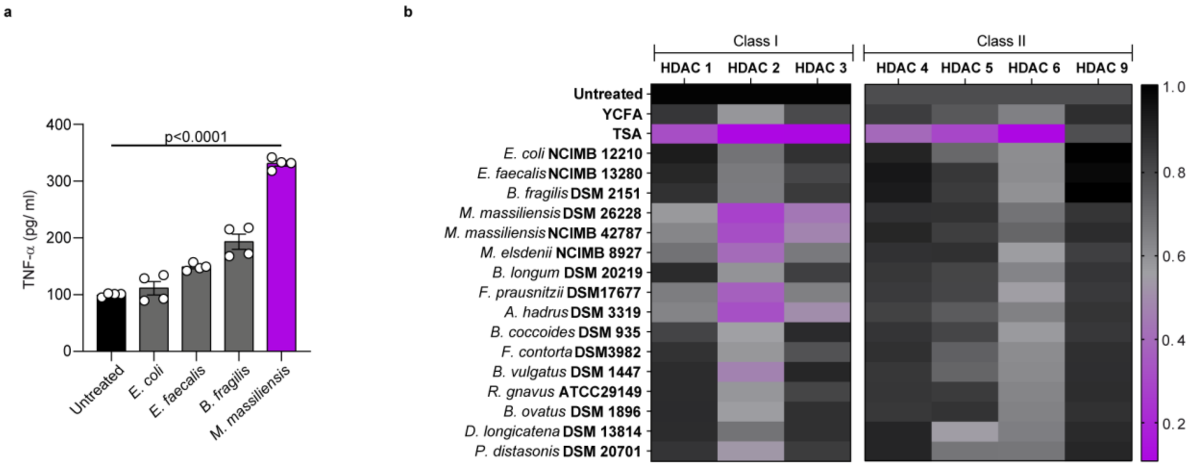

Supplementary Figure 1. Influence of commensal-derived supernatants on TNF-α secretion and HDAC activity. a, The secretion of TNF-α from murine CTLs was measured by ELISA after three days of stimulation with supernatants of indicated bacteria (n = 4 mice, 1:20 supernatant-to-cell media ratio for all examined bacteria, two-tailed unpaired Student's *t*-test was performed; results are shown as mean ± s.e.m). **b**, HDAC inhibition of recombinant class I and class II HDAC isoforms by cell-free supernatants derived from 16 members of human commensal community. Significance was tested against YCFA medium. One of three similar experiments is shown. Source data are provided as a Source Data file.

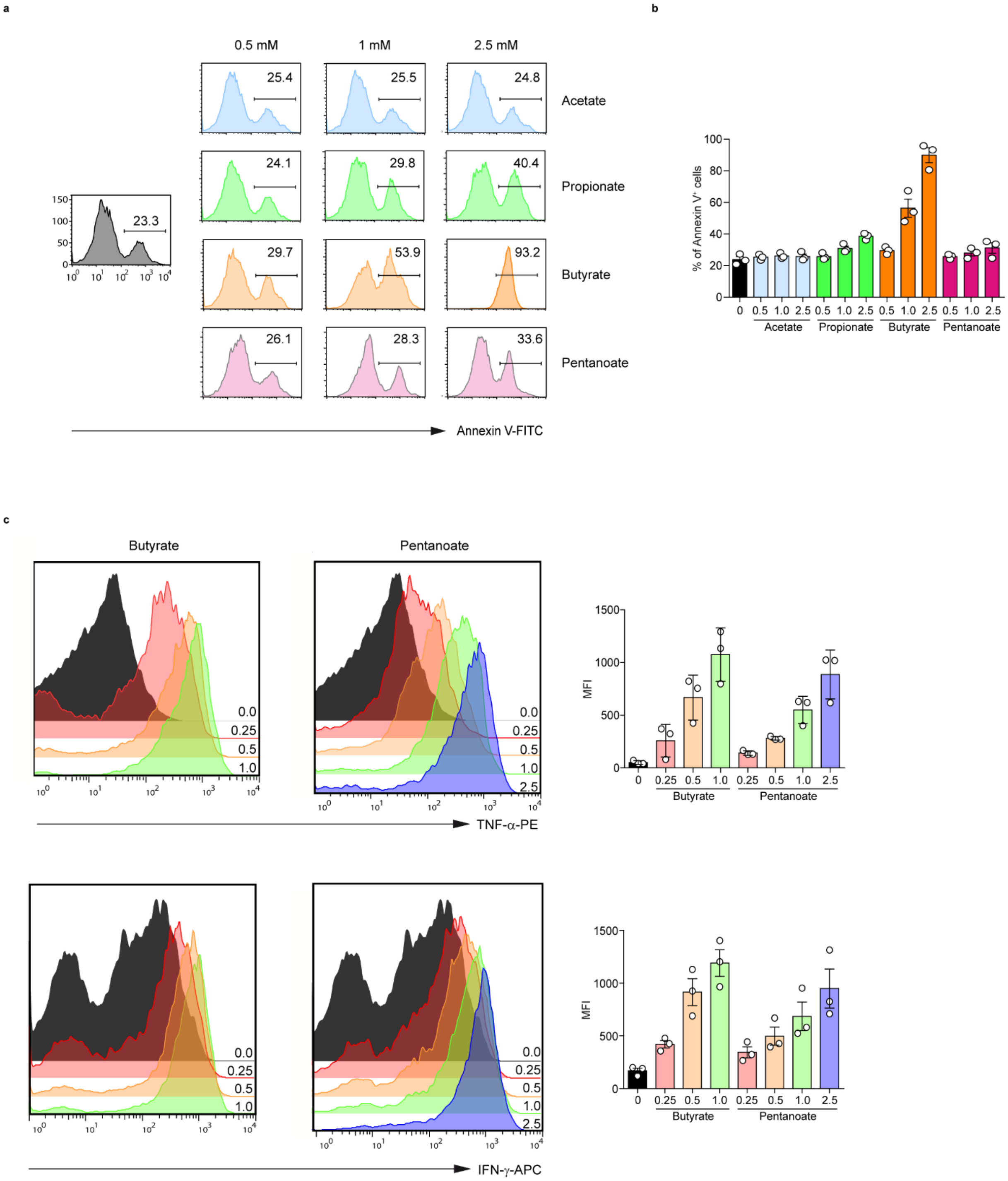

Supplementary Figure 2. Impact of SCFAs on the apoptosis and cytokine production in CTLs. a,**b**, Murine CD8^{+} T cells were polarized under CTL-inducing conditions for three days in the presence of indicated SCFA concentrations. Representative histogram plots show the frequency of Annexin V^{+} cells (**a**). The bar graphs (**b**) show the quantification of apoptosis in CTLs treated with SCFAs (n = 3 independent experiments). **c**, Murine CTLs were cultured under suboptimal conditions and treated with increasing conentrations of butyrate and pentanoate, respectively. The representative histograms (left) show the expression of TNF-α and IFN-γ, measured by flow cytometry. The bar graphs (right) display the mean fluorescence intensity (MFI) values of TNF-α and IFN-γ (n = 3 independent experiments). Results in **b, c** are shown as mean ± s.e.m. Source data are provided as a Source Data file.

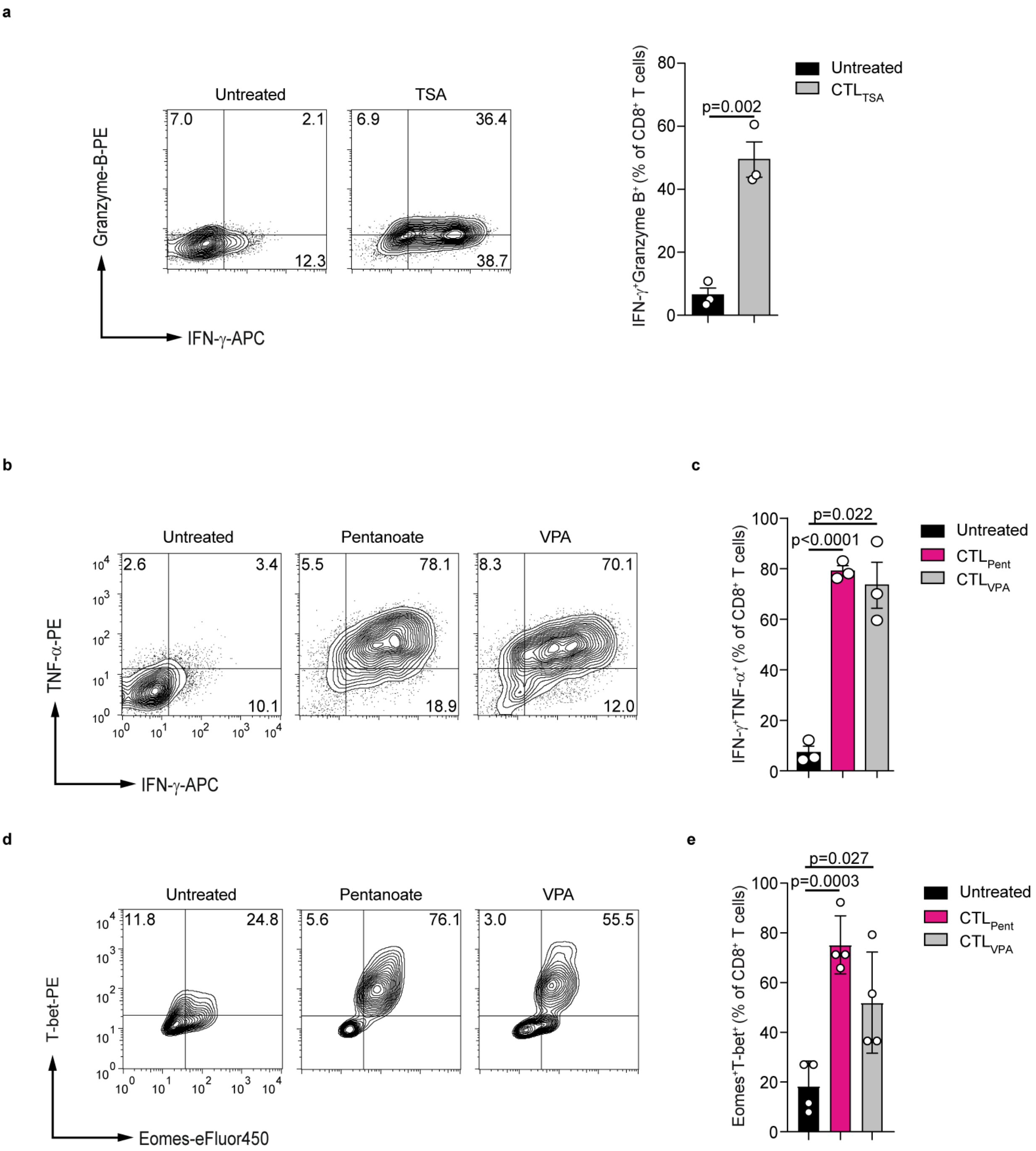

Supplementary Figure 3. Pentanoate induces the expression of CTL-associated molecules via HDAC-inhibitory activity. a-e, $CD8^+$ T cells were polarized under suboptimal CTL-inducing conditions in absence or presence of pentanoate (2.5 mM), valproate (VPA, 0.5 mM) or TSA (10 nM) for three days. In (**a**), representative contour plots and bar graphs show the frequency of IFN-γ^+ and granzyme B^+ cells (n = 3 independent experiments). The contour plots and bar graphs indicate the percentage of IFN-γ^+TNF-α^+ (**b**,**c**) or T-$bet^+$$Eomes^+$ (**d**,**e**) cells (in **c**, n = 3 independent experiments; in **e**, n = 4 independent experiments). Statistical analysis was performed by two-tailed unpaired Student's *t*-test; results are shown as mean ± s.e.m. Source data are provided as a Source Data file.

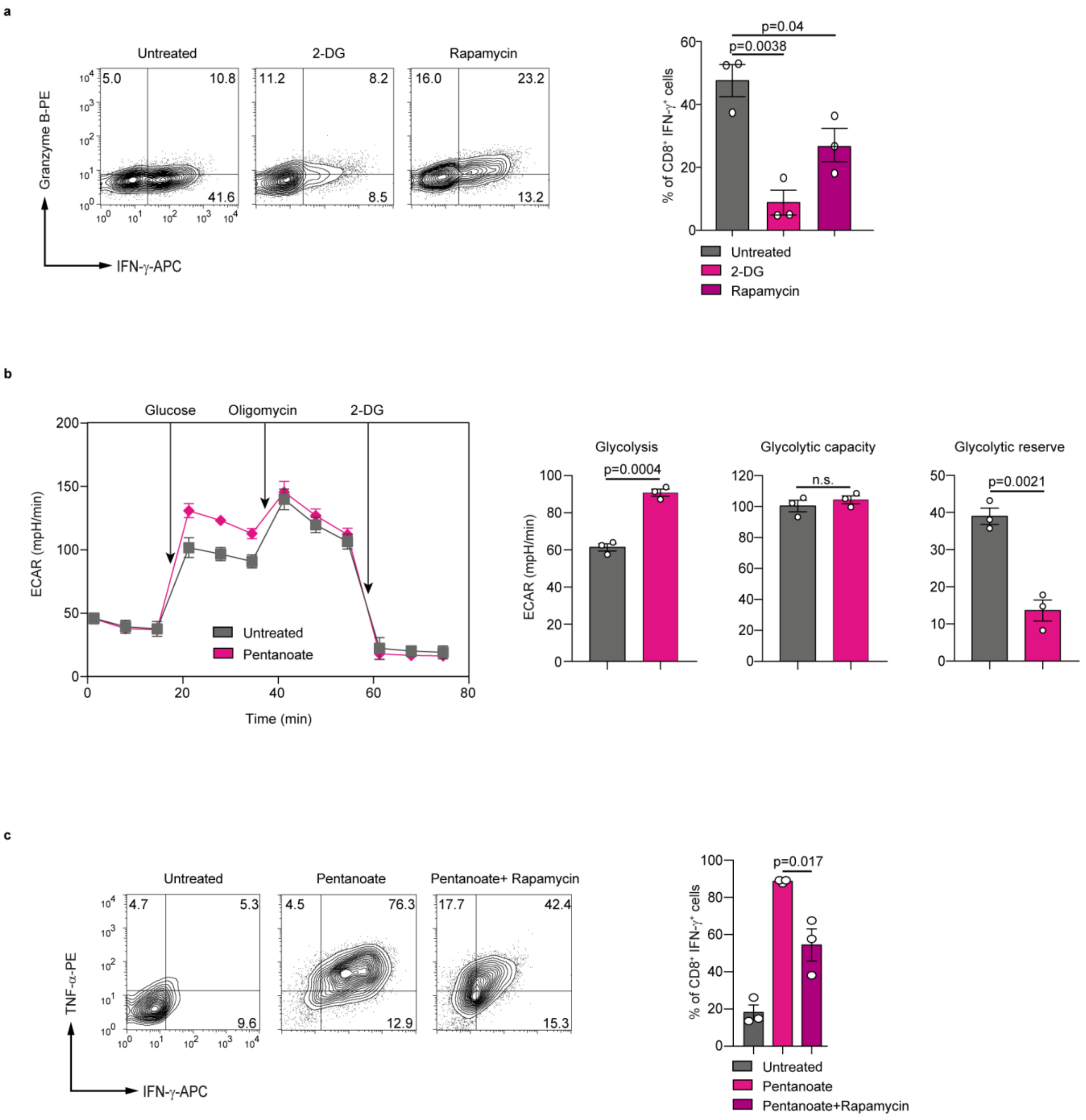
a
Untreated
2-DG
Rapamycin
Granzyme B-PE
IFN-γ-APC
5.0
10.8
41.6
11.2
8.2
8.5
16.0
23.2
13.2
% of CD8+ IFN-γ+ cells
p=0.04
p=0.0038
Untreated
2-DG
Rapamycin
b
Glucose
Oligomycin
2-DG
ECAR (mpH/min)
Time (min)
Untreated
Pentanoate
Glycolysis
p=0.0004
Glycolytic capacity
n.s.
Glycolytic reserve
p=0.0021
ECAR (mpH/min)
c
Untreated
Pentanoate
Pentanoate+ Rapamycin
TNF-α-PE
IFN-γ-APC
4.7
5.3
9.6
4.5
76.3
12.9
17.7
42.4
15.3
% of CD8+ IFN-γ+ cells
p=0.017
Untreated
Pentanoate
Pentanoate+Rapamycin

Supplementary Figure 4. Pentanoate enhances the mTOR activity in CTLs. a, Murine $CD8^+$ T cells were polarized under suboptimal CTL-inducing conditions for three days. Cells were polarized in presence or absence of 2-DG (1 mM) or rapamycin (100 nM), respectively. Representative contour plots indicate the frequency of granzyme B^+ and IFN-γ^+ cells (n = 3 independent experiments). **b**, Measurement of extracellular acidification rate (ECAR) for *in vitro* generated murine CTLs cultured with or without 2.5 mM pentanoate for three days. ECAR was measured under basal conditions and in response to glucose (10 mM), oligomycin (2 μM), and 2-deoxy-glucose (2-DG, 100 mM). Quantification of glycolysis, glycolytic capacity and glycolytic reserve combined from three experiments is shown (right). **c**, $CD8^+$ T cells were polarized under suboptimal CTL-inducing conditions for three days. Cells were treated with pentanoate in presence or absence of rapamycin or left untreated. Representative contour plots and bar graphs indicate the frequency of TNF-α^+ and IFN-γ^+ cells (n = 3 independent experiments, n.s. = not significant). Statistical analysis was performed by two-tailed unpaired Student's *t*-test; results are shown as mean ± s.e.m. Source data are provided as a Source Data file.

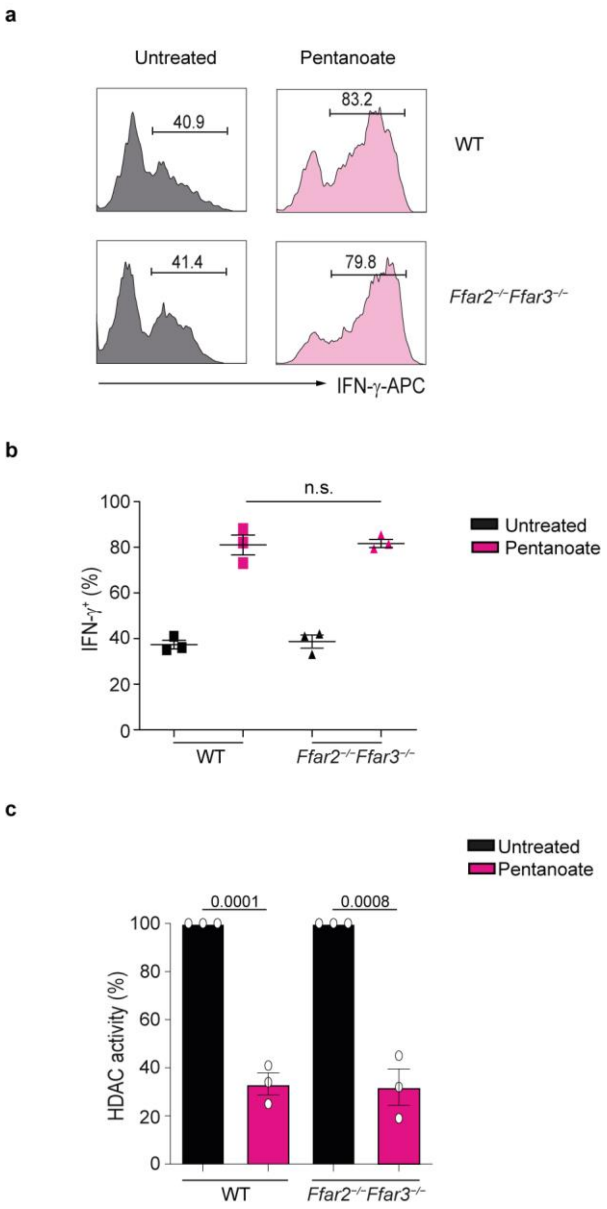

Supplementary Figure 5. Impact of SCFA-receptors GPR41 and GPR43 on the pentanoate-mediated modulation of CTLs. a,**b**, Following the treatment of *Ffar2*$^{-/-}$*Ffar3*$^{-/-}$ (deficient for GPR41 and GPR43) and WT CTLs with 2.5 mM pentanoate for three days, the percentage of IFN-γ^{+}CD8^{+} T cells was measured by FACS analysis. Three independent experiments were performed. Results (**b**) are expressed as mean ± SEM (n. s. = not significant). **c**, The fluorogenic HDAC activity assay was carried out to measure the HDAC activity in CTL-derived cell lysates obtained after three days of the cell culture. The assay was performed in the presence or absence of 5 mM pentanoate. The HDAC activity measured for untreated CTL-derived cell lysates was arbitrary set at 100 percent (n = 3 independent experiments). Statistical analysis was performed by two-tailed unpaired Student's *t*-test; results are shown as mean ± s.e.m. Source data are provided as a Source Data file.

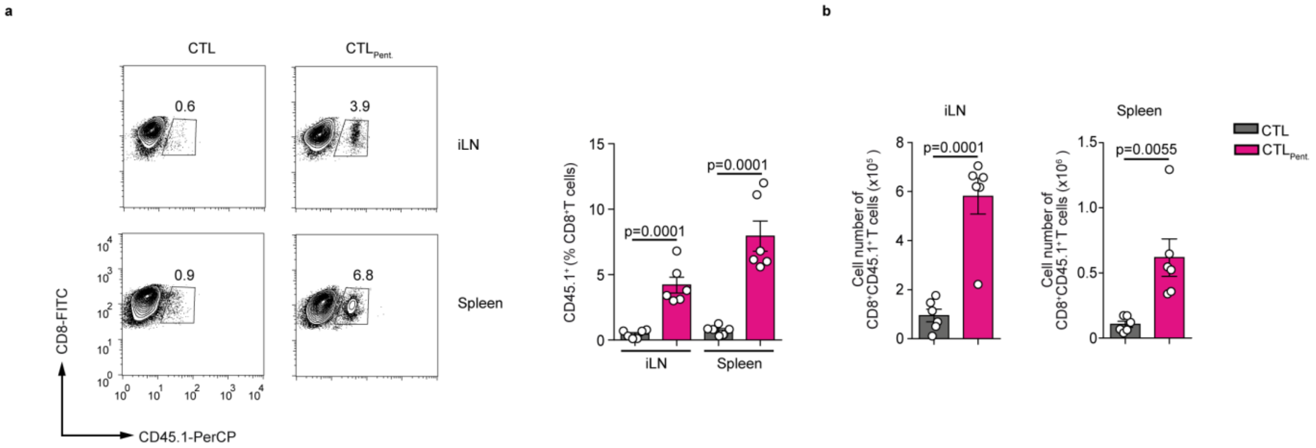

Supplementary Figure 6. Pentanoate promotes persistence of antigen-specific CTLs. a,**b**, After three days of pretreatment with pentanoate, CD45.1$^+$ OVA-specific CTLs were transferred intraperitoneally (i.p.) into CD45.2$^+$ animals bearing 5-days old PancOVA tumors. The frequencies (**a**) and total cell numbers (**b**) of CD45.1$^+$ tumor-specific CTLs were measured by FACS analysis in draining LNs (iLNs) and spleens on day 23 post tumor inoculation (n = 6 mice/group combined from 2 independent experiments**,** the groups were analysed by two-tailed unpaired Student's *t*-test, results are shown as mean ± s.e.m.). Source data are provided as a Source Data file.

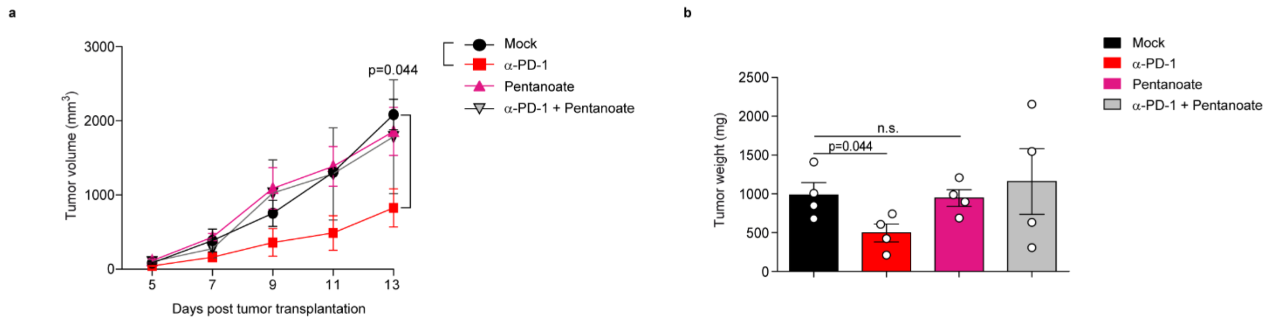

Supplementary Figure 7. Combination of pentanoate and anti-PD-1 does not enhance the efficacy of immune checkpoint inhibition. a,**b**, 8- to 12-week-old C57BL/6N female mice were inoculated subcutaneously with 1×10^6 B16F10 tumor cells at the right flank. Mice were i.p. treated with 10 mg/kg anti-PD-1 antibody (clone J43) on days 6, 9 and 12. Two groups of mice were additionally treated with pentanoate by i.p. injection every other day at 500 mg per kg body weight. One of two independent experiments is displayed (n = 4 mice per group/ experiment, n.s. = not significant). Representative tumor growth curves (**a**) and tumor weight on day 13 after inocculation of tumor cells (**b**) are shown. Statistical analysis was performed by two-tailed unpaired Student's *t*-test; results are shown as mean ± s.e.m. Source data are provided as a Source Data file.

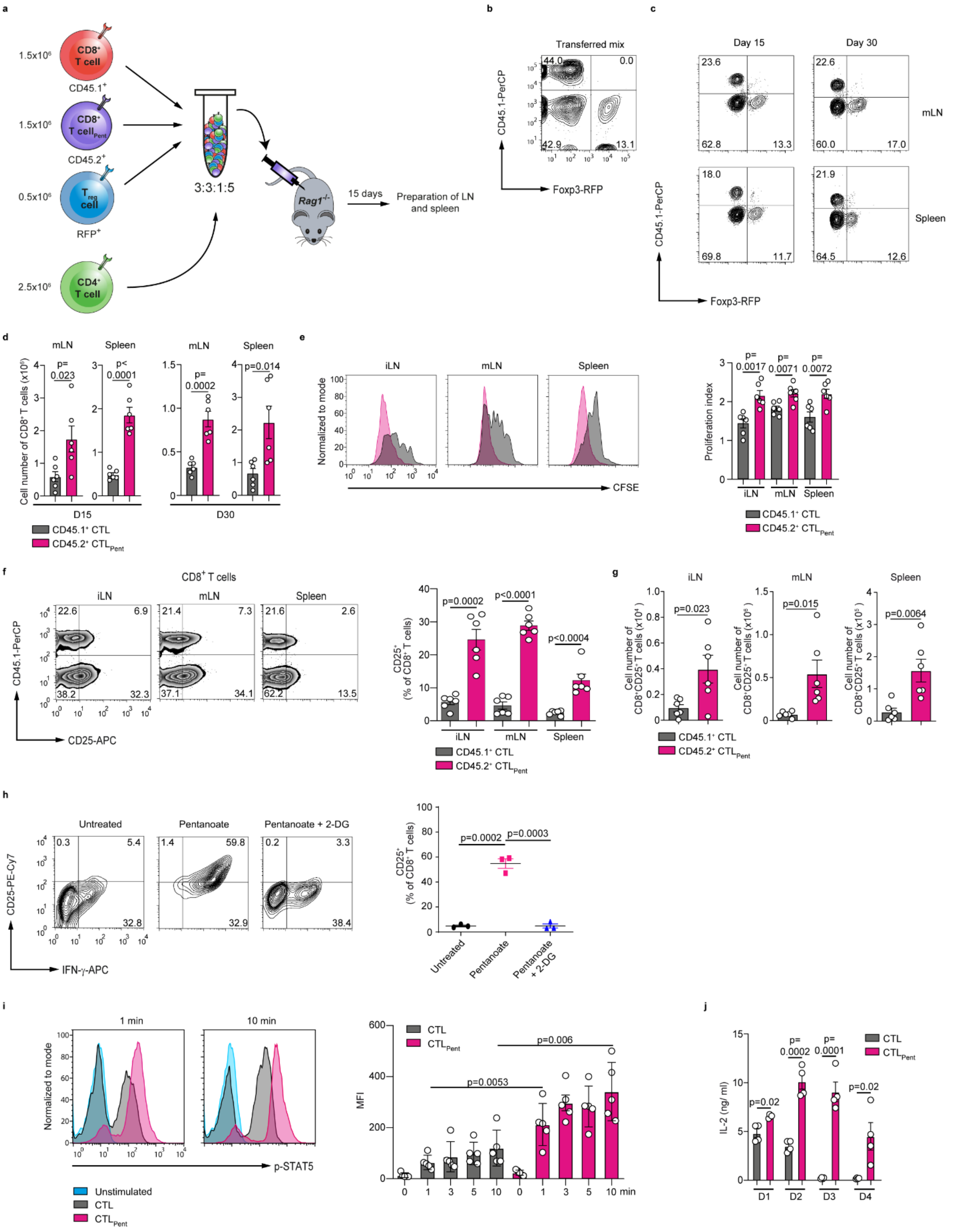
a
1.5x10^6
CD8+ T cell
CD45.1+
1.5x10^6
CD8+ T cellPent
CD45.2+
0.5x10^6
Treg cell
RFP+
2.5x10^6
CD4+ T cell
3:3:1:5
Rag1-/-
15 days
Preparation of LN and spleen
b
Transferred mix
44.0
0.0
42.9
13.1
CD45.1-PerCP
Foxp3-RFP
c
Day 15
Day 30
23.6
62.8
13.3
22.6
60.0
17.0
mLN
18.0
69.8
11.7
21.9
64.5
12.6
Spleen
CD45.1-PerCP
Foxp3-RFP
d
mLN
Spleen
mLN
Spleen
p=0.023
p<0.0001
p=0.0002
p=0.014
Cell number of CD8+ T cells (x10^6)
D15
D30
CD45.1+ CTL
CD45.2+ CTLPent
e
iLN
mLN
Spleen
Normalized to mode
CFSE
p=0.0017
p=0.0071
p=0.0072
Proliferation index
iLN
mLN
Spleen
CD45.1+ CTL
CD45.2+ CTLPent
f
CD8+ T cells
iLN
mLN
Spleen
22.6
6.9
38.2
32.3
21.4
7.3
37.1
34.1
21.6
2.6
62.2
13.5
CD45.1-PerCP
CD25-APC
p=0.0002
p<0.0001
p<0.0004
CD25+ (% of CD8+ T cells)
iLN
mLN
Spleen
CD45.1+ CTL
CD45.2+ CTLPent
g
iLN
mLN
Spleen
p=0.023
p=0.015
p=0.0064
Cell number of CD8+CD25+ T cells (x10^4)
Cell number of CD8+CD25+ T cells (x10^5)
Cell number of CD8+CD25+ T cells (x10^5)
CD45.1+ CTL
CD45.2+ CTLPent
h
Untreated
Pentanoate
Pentanoate + 2-DG
0.3
5.4
32.8
1.4
59.8
32.9
0.2
3.3
38.4
CD25-PE-Cy7
IFN-γ-APC
p=0.0002
p=0.0003
CD25+ (% of CD8+ T cells)
Untreated
Pentanoate
Pentanoate + 2-DG
i
1 min
10 min
Normalized to mode
p-STAT5
Unstimulated
CTL
CTLPent
CTL
CTLPent
p=0.006
p=0.0053
MFI
0
1
3
5
10
0
1
3
5
10 min
j
CTL
CTLPent
p=0.0002
p=0.0001
p=0.02
p=0.02
IL-2 (ng/ ml)
D1
D2
D3
D4

Supplementary Figure 8. Pentanoate enhances expression of CD25 and IL-2 in CTLs. a,b, Experimental design for the analysis of *in vivo* persistence of CTLs pretreated with pentanoate ($CD8^+$ T $cell_{Pent}$) in the absence of antigen. **c**,**d**, The frequency (**c**) and total cell numbers (**d**) of transferred T cells (WT $CD45.1^+$ CTLs, WT $CD45.2^+$ pentanoate-treated CTLs and $Foxp3^+CD45.2^+$ Tregs from *FIR* × *tiger* mice) in Rag1-deficient mice on days 15 and 30 after the adoptive transfer. The co-transferred $Foxp3^-CD4^+$ cells were excluded from the gate (**b,c**). **e**, CFSE staining for pentanoate-pretreated CTLs on day 15 after adoptive transfer into Rag-1 deficient mice. On the right side, the proliferation index (the total number of divisions divided by the number of CTLs that went into division) is shown. **f**, **g**, Frequencies and cell numbers of $CD25^+CD8^+$ T cells were analyzed by flow cytometry on day 15 after transfer of control CTLs ($CD45.1^+$) and pentanoate-treated CTLs ($CD45.2^+$) into Rag1-deficient mice (the experimental setting is described in **a**,**b)**. Data (**d-g**) were analyzed by two-tailed unpaired Student's *t*-test (mean ± s.e.m, n = 6 mice combined from 2 independent experiments). **h**, The percentage of murine $CD25^+IFN\text{-}\gamma^+$CTLs treated with pentanoate (2.5 mM), or pentanoate (2.5 mM) + 2-DG (1 mM) for three days (n = 3 mice**,** analysed by two-tailed unpaired Student's *t*-test; data are shown as mean ± s.e.m). **i**, Murine $CD8^+$ T cells were differentiated into CTLs in the presence or absence of pentanoate for 3 day, then washed and rested for 4 hours. Subsequently, cells were treated with IL-2 (50 U/ml) for indicated time points. The phosphorylated (p)-STAT5 levels were analyzed by flow cytometry (n = 5 mice, data are mean ± s.e.m, two-tailed unpaired Student's *t*-test. **j**, The secretion of IL-2 in pentanoate-treated CTLs cultured for indicated time points was measured by ELISA (n = 4 mice, two-tailed unpaired Student's *t*-test was performed; results are shown as mean ± s.e.m). Source data are provided as a Source Data file.

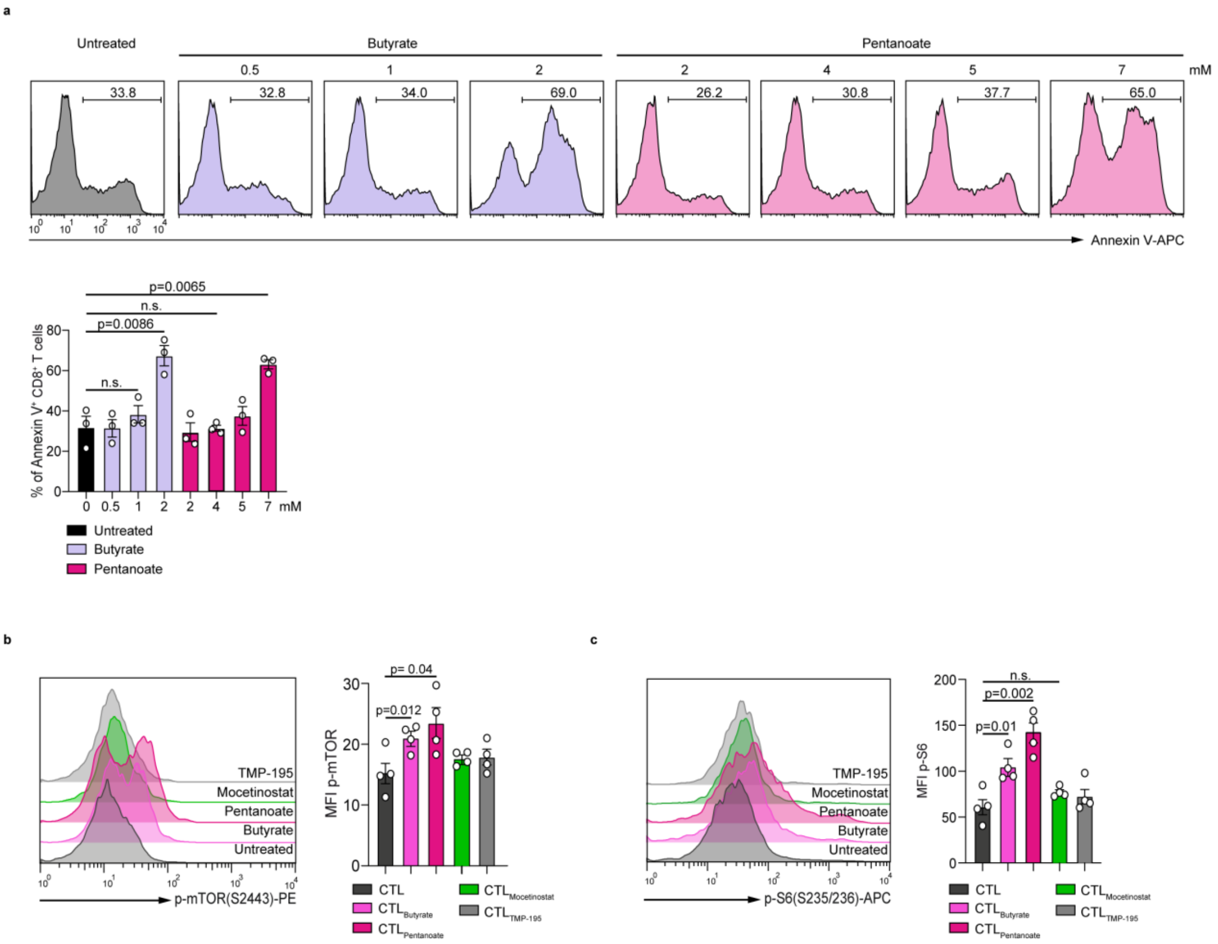

Supplementary Figure 9. SCFAs pentanoate and butyrate enhance the activity of mTOR in human $CD8^+$ T cells. a, Human $CD8^+$ T cells were cultured for three days under CTL-conditions in the presence of increassing butyrate or pentanoate concentrations. The percentage of Annexin V^+ cells was measured by flow cytometry. Three independent experiments were performed (n.s. = not significant; two-tailed Student's *t*-test was performed; results are shown as mean ± s.e.m). **b**,**c**, $CD8^+$ T cells isolated from peripheral blood of healthy donors were differentiated into CTLs in presence or absence of indicated HDACi (2.5 µM TMP-195, 300 nM mocetinostat, 4 mM pentanoate, 1 mM butyrate). Representative histogram plots and bar graphs indicate the phoshorylated levels of mTOR (**b**) and S6 ribosomal protein (**c**) (n = 4 donors, the groups were compared by two-tailed Student's *t*-test; data are shown as mean ± s.e.m, n.s. = not siginficant). Source data are provided as a Source Data file.

Gating strategies

a

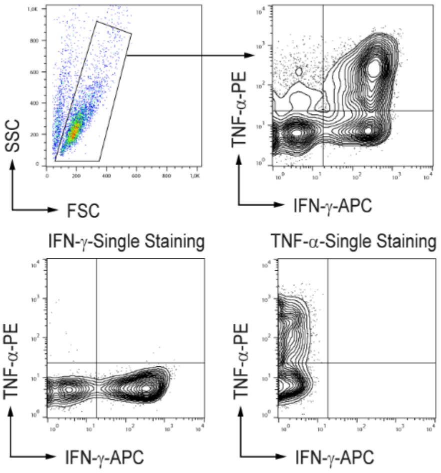

b

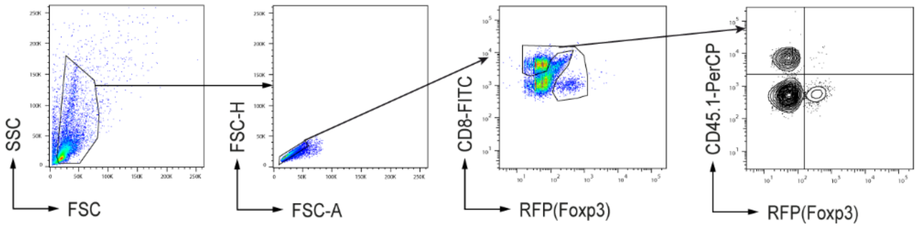

c

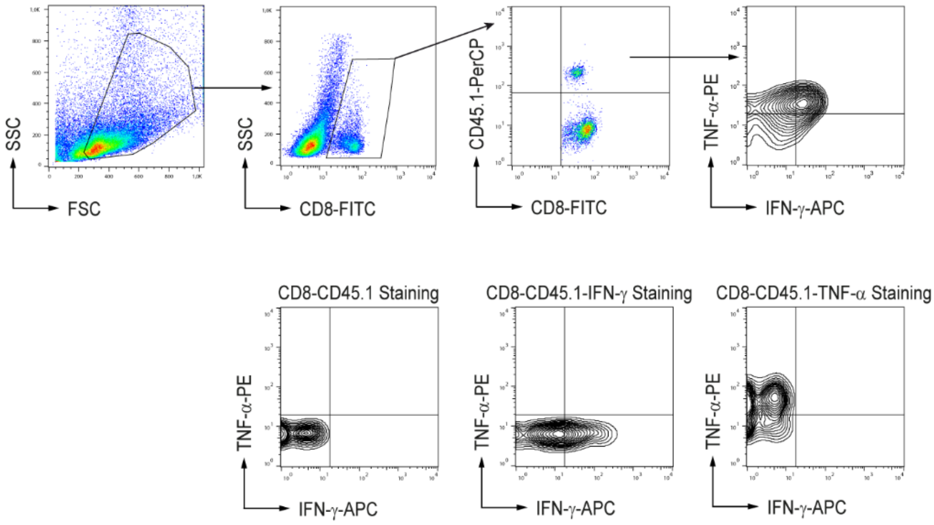

d

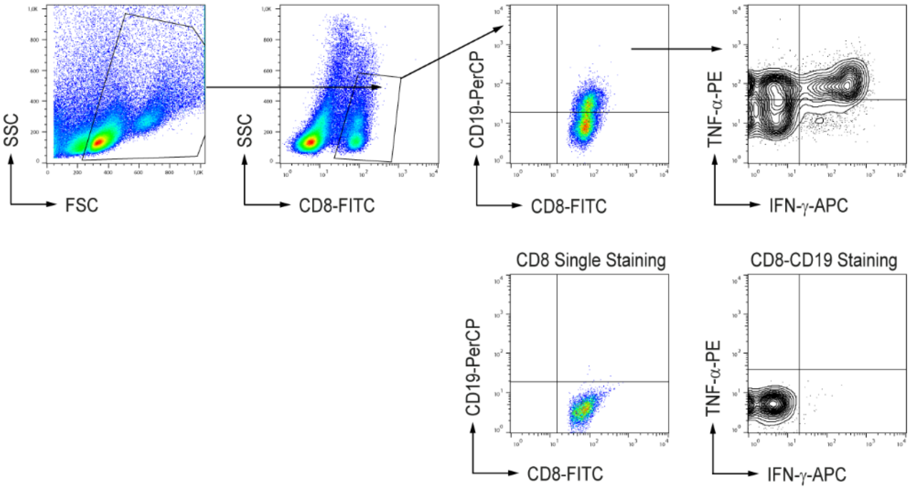

Supplementary Figure 10. Gating strategies for FACS analysis of T cells. a-c, Different gating strategies (including control stainings for FACS analysis of effector cytokines) for $CD8^+$ T cells and Tregs are shown. **d**, Gating strategy for the flow cytometry analysis of $CD19^{t+}$ CAR T cells recognizing ROR1.

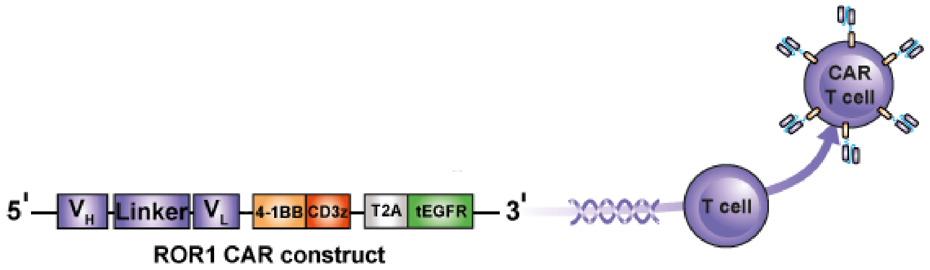

Supplementary Figure 11. Human CAR construct. A cartoon illustrating the design and structure of the human CAR construct used in this study.
