# Supplementary material for: Microbial short-chain fatty acids modulate CD8+ T cell responses and improve adoptive immunotherapy for cancer
Source: Nat Commun. 2021 Jul 1;12:4077. doi: 10.1038/s41467-021-24331-1 (PMC8249424; doi:10.1038/s41467-021-24331-1)
Supplement: Supplementary file 3 — Reporting Summary [file 41467_2021_24331_MOESM3_ESM.pdf]

nature research

Corresponding author(s): Alexander Visekruna, Michael Hudecek

Last updated by author(s): May 25, 2021

Reporting Summary

Nature Research wishes to improve the reproducibility of the work that we publish. This form provides structure for consistency and transparency in reporting. For further information on Nature Research policies, see our Editorial Policies and the Editorial Policy Checklist.

Statistics

For all statistical analyses, confirm that the following items are present in the figure legend, table legend, main text, or Methods section.

n/a	Confirmed	
☐	☒	The exact sample size (*n*) for each experimental group/condition, given as a discrete number and unit of measurement
☐	☒	A statement on whether measurements were taken from distinct samples or whether the same sample was measured repeatedly
☐	☒	The statistical test(s) used AND whether they are one- or two-sided<br>*Only common tests should be described solely by name; describe more complex techniques in the Methods section.*
☐	☒	A description of all covariates tested
☐	☒	A description of any assumptions or corrections, such as tests of normality and adjustment for multiple comparisons
☐	☒	A full description of the statistical parameters including central tendency (e.g. means) or other basic estimates (e.g. regression coefficient) AND variation (e.g. standard deviation) or associated estimates of uncertainty (e.g. confidence intervals)
☐	☒	For null hypothesis testing, the test statistic (e.g. *F*, *t*, *r*) with confidence intervals, effect sizes, degrees of freedom and *P* value noted<br>*Give P values as exact values whenever suitable.*
☒	☐	For Bayesian analysis, information on the choice of priors and Markov chain Monte Carlo settings
☒	☐	For hierarchical and complex designs, identification of the appropriate level for tests and full reporting of outcomes
☒	☐	Estimates of effect sizes (e.g. Cohen's *d*, Pearson's *r*), indicating how they were calculated

Our web collection on statistics for biologists contains articles on many of the points above.

Software and code

Policy information about availability of computer code

Data collection	Cytometry: Cell Quest Pro version 5.1, BD FACSDiva 6.1.3<br>Plate reader assays: Omega series Software V5.5, MARS 3.32 R5
Data analysis	Cytometry: FlowJo V10<br>Plate reader assays: Omega series Software V5.5, MARS 3.32 R5<br>Statistics: GraphPad Prism 8

For manuscripts utilizing custom algorithms or software that are central to the research but not yet described in published literature, software must be made available to editors and reviewers. We strongly encourage code deposition in a community repository (e.g. GitHub). See the Nature Research guidelines for submitting code & software for further information.

Data

Policy information about availability of data

All manuscripts must include a data availability statement. This statement should provide the following information, where applicable:

- Accession codes, unique identifiers, or web links for publicly available datasets
- A list of figures that have associated raw data
- A description of any restrictions on data availability

The authors declare that data supporting the findings of this study are available within the paper and its supplementary information files.

Field-specific reporting

Please select the one below that is the best fit for your research. If you are not sure, read the appropriate sections before making your selection.

- [x] Life sciences
- [ ] Behavioural & social sciences
- [ ] Ecological, evolutionary & environmental sciences

For a reference copy of the document with all sections, see nature.com/documents/nr-reporting-summary-flat.pdf

Life sciences study design

All studies must disclose on these points even when the disclosure is negative.

Sample size	Although statistical methods were not used to predetermine sample size, sample sizes were chosen on the basis of estimates from pilot experiments. Sample sizes are similar to those generally employed and accepted in the field. All sample sizes are shown in the figures legends or in the Methods section of our manuscript, respectively. In the in vivo experiments, numbers of animals are similar to those generally used in similar studies, and were sufficient to support our conclusions with statistical significance.
Data exclusions	No data were excluded from analysis.
Replication	All results obtained in this study were successfully replicated. We have performed the experiments shown in our manuscript at least twice, in most cases even more than twice. Exact numbers of biologically independent repetitions are stated in the manuscript.
Randomization	To collect unbiased data, animals were randomly asigned to the mock or SCFA-treated group. Mice of the same sex and age were used to control for covariates. Blood samples from healthy donors were obtained randomized from the University Hospital Würzburg and were not allocated into experimental groups.
Blinding	In vivo experiments were performed unblinded due to the requirements and regulations of the animal facility BMFZ and the Regierungspräsidium Gießen, Germany. However, at least two scientists performed the experiments and analyzed the described data independently. In in vitro experiments, blinding was not necessary since all the samples were analyzed in the same way.

Reporting for specific materials, systems and methods

We require information from authors about some types of materials, experimental systems and methods used in many studies. Here, indicate whether each material, system or method listed is relevant to your study. If you are not sure if a list item applies to your research, read the appropriate section before selecting a response.

Materials & experimental systems

n/a	Involved in the study	
[ ]	[x]	Antibodies
[ ]	[x]	Eukaryotic cell lines
[x]	[ ]	Palaeontology and archaeology
[ ]	[x]	Animals and other organisms
[ ]	[x]	Human research participants
[x]	[ ]	Clinical data
[x]	[ ]	Dual use research of concern

Methods

n/a	Involved in the study	
[x]	[ ]	ChIP-seq
[ ]	[x]	Flow cytometry
[x]	[ ]	MRI-based neuroimaging

Antibodies

Antibodies used

All antibodies used for cytometry are from commercial sources and were purchased from Biolegend, eBioscience, BD Pharmingen/ Bioscience, Invitrogen or Miltenyi Biotech.

Anti-mouse:
CD3, clone 145-2C11 (Cat# 100312 - Biolegend, Cat# 100306 - Biolegend, Cat# 100302 - Biolegend)
CD28, clone 37.51 (Cat# 102116 - Biolegend)
CD4, clone RM4-5 (Cat# 100510 - Biolegend, Cat# 100538 - Biolegend, Cat# 560468 - BD)
CD8, clone 53-6.7 (Cat# 100706 - Biolegend, Cat# 100712 - Biolegend, Cat# 560776 - BD)
CD45.1, clone A20 (Cat# 110726 - Biolegend)
CD25, clone PC61.5 (Cat# 102012 - 110726, Cat# 102016 - 110726)
CD19, clone 1D3/CD19 (Cat# 152406 - Biolegend)
IFN-gamma, clone XMG1.2 (Cat# 505810 - Biolegend, Cat# 16-7311-85 - Invitrogen)
TNF-alpha, clone MP6-XT22 (Cat# 506304 - Biolegend, Cat# Biolegend)
Granzyme B, clone QA16A02 (Cat# 372208 - Biolegend)
Eomes, clone Dan11mag, (Cat# 48-4875-82 - eBioscience)

T-bet, clone eBio4B10 (Cat# 12-5825-82 - eBioscience)
phospho-STAT5 (Tyr694), clone SRBCZX (Cat# 12-9010-42 - eBioscience)
phospho-mTOR (ser2448), clone MRRBY (Cat# 12-9718-42 - eBioscience)
phospho-S6 (Ser235/236), clone cupk43k (Cat# 17-9007-42 - eBioscience)

Anti-human:
IFN-gamma, clone 4S.B3 (Cat# 502512 - Biolegend)
Granzyme B, clone QA16A02 (Cat# 372208 - Biolegend)
CD25, clone M-A251 (Cat# 356106 - Biolegend)
CD69, clone FN50 (Cat# 310914 - Biolegend)
CD8, clone SK1 (Cat# 561618 - BD)

For the analysis of secreted cytokines, following commercial kits were used:

BioLegend ELISA MAX Deluxe Set Human IFN-y (Cat# 430104)
BioLegend ELISA MAX Deluxe Set Human IL-2 (Cat# 431804)
BioLegend ELISA MAX Deluxe Set Human TNF-a (Cat# 430204)
Invitrogen Murine TNF-alpha ELISA Kit (Cat# 88-7324-76)
Biolegend Max Standard Set Mouse IL-2 (Cat# 431001)

Validation

No customized antibodies were used. Validation data of the antibodies purchased from commercial vendors are available on the manufactures' website and datasheets.

Anti-mouse:
CD3, clone 145-2C11 (Cat# 100312 - Biolegend, Cat# 100306 - Biolegend, Cat# 100302 - Biolegend)
https://www.biolegend.com/en-us/products/apc-anti-mouse-cd3epsilon-antibody-21
https://www.biolegend.com/en-us/products/fitc-anti-mouse-cd3epsilon-antibody-23
https://www.biolegend.com/en-us/products/purified-anti-mouse-cd3epsilon-antibody-28?GroupID=BLG6744

CD28, clone 37.51 (Cat# 102116 - Biolegend)
https://www.biolegend.com/en-us/products/ultra-leaf-purified-anti-mouse-cd28-antibody-7733

CD4, clone RM4-5 (Cat# 100510 - Biolegend, Cat# 100538 - Biolegend, Cat# 560468 - BD)
https://www.biolegend.com/en-us/products/fitc-anti-mouse-cd4-antibody-480?GroupID=BLG4745
https://www.biolegend.com/en-us/products/percp-anti-mouse-cd4-antibody-4229?GroupID=BLG4211
https://www.bdbiosciences.com/eu/applications/research/stem-cell-research/hematopoietic-stem-cell-markers/mouse/negative-markers/v450-rat-anti-mouse-cd4-rm4-5-also-known-as-rm45/p/560468

CD8, clone 53-6.7 (Cat# 100706 - Biolegend, Cat# 100712 - Biolegend, Cat# 560776 - BD)
https://www.biolegend.com/en-us/products/fitc-anti-mouse-cd8a-antibody-153?GroupID=BLG2559
https://www.biolegend.com/en-us/search-results/apc-anti-mouse-cd8a-antibody-150
https://www.bdbiosciences.com/eu/reagents/research/antibodies-buffers/immunology-reagents/anti-mouse-antibodies/cell-surface-antigens/v500-rat-anti-mouse-cd8a-53-67/p/560776

CD45.1, clone A20 (Cat# 110726 - Biolegend)
https://www.biolegend.com/en-us/global-elements/pdf-popup/percp-anti-mouse-cd45-1-antibody-4268?GroupID=BLG1933

CD25, clone PC61.5 (Cat# 102012 - Biolegend, Cat# 102016 - Biolegend)
https://www.biolegend.com/fr-ch/products/apc-anti-mouse-cd25-antibody-420
https://www.biolegend.com/en-gb/sean-tuckers-tests/pe-cyanine7-anti-mouse-cd25-antibody-1929?GroupID=BLG10428

CD19, clone 1D3/CD19 (Cat# 152406 - Biolegend)
https://www.biolegend.com/en-us/products/percp-cyanine5-5-anti-mouse-cd19-antibody-13640?GroupID=BLG15527

IFN-gamma, clone XMG1.2 (Cat# 505810 - Biolegend, Cat# 16-7311-85 - Invitrogen)
https://www.biolegend.com/en-us/search-results/apc-anti-mouse-ifn-gamma-antibody-993
https://www.thermofisher.com/antibody/product/IFN-gamma-Antibody-clone-XMG1-2-Monoclonal/16-7311-81

TNF-alpha, clone MP6-XT22 (Cat# 506304 - Biolegend, Cat# 506306 - Biolegend)
https://www.biolegend.com/en-us/search-results/fitc-anti-mouse-tnf-alpha-antibody-976
https://www.biolegend.com/en-us/products/pe-anti-mouse-tnf-alpha-antibody-978

Granzyme B, clone QA16A02 (Cat# 372208 - Biolegend)
https://www.biolegend.com/en-us/products/pe-anti-human-mouse-granzyme-b-recombinant-antibody-14431?GroupID=GROUP28

Eomes, clone Dan11mag, (Cat# 48-4875-82 - eBioscience)

https://www.thermofisher.com/antibody/product/EOMES-Antibody-clone-Dan11mag-Monoclonal/48-4875-82

T-bet, clone eBio4B10 (Cat# 12-5825-82 - eBioscience)
https://www.thermofisher.com/antibody/product/T-bet-Antibody-clone-eBio4B10-4B10-Monoclonal/12-5825-82

phospho-STAT5 (Tyr694), clone SRBCZX (Cat# 12-9010-42 - eBioscience)
https://www.thermofisher.com/antibody/product/Phospho-STAT5-Tyr694-Antibody-clone-SRBCZX-Monoclonal/12-9010-42

phospho-mTOR (ser2448), clone MRRBY (Cat# 12-9718-42 - eBioscience)
https://www.thermofisher.com/antibody/product/Phospho-mTOR-Ser2448-Antibody-clone-MRRBY-Monoclonal/12-9718-42

phospho-S6 (Ser235/236), clone cupk43k (Cat# 17-9007-42 - eBioscience)
https://www.thermofisher.com/antibody/product/Phospho-S6-Ser235-Ser236-Antibody-clone-cupk43k-Monoclonal/17-9007-42

Anti-human:
IFN-gamma, clone 4S.B3 (Cat# 502512 - Biolegend)
https://www.biolegend.com/en-gb/products/apc-anti-human-ifn-gamma-antibody-1012

Granzyme B, clone QA16A02 (Cat# 372208 - Biolegend)
https://www.biolegend.com/en-us/products/pe-anti-human-mouse-granzyme-b-recombinant-antibody-14431?GroupID=GROUP28

CD25, clone M-A251 (Cat# 356106 - Biolegend)
https://www.biolegend.com/en-us/products/fitc-anti-human-cd25-antibody-8390

CD69, clone FN50 (Cat# 310914 - Biolegend)
https://www.biolegend.com/en-gb/products/apc-cyanine7-anti-human-cd69-antibody-1917

CD8, clone SK1 (Cat# 561618 - BD)
https://www.bdbiosciences.com/us/reagents/research/antibodies-buffers/immunology-reagents/anti-non-human-primate-antibodies/cell-surface-antigens/v500-mouse-anti-human-cd8-sk1/p/561618

Eukaryotic cell lines

Policy information about cell lines

Cell line source(s)	K562 (ATCC, Manassas, VA, USA)<br>K562_ROR1 were generated by lentiviral transduction with the full-length human ROR1 gene. All tumor cell lines were transduced with a lentiviral vector encoding firefly luciferase (ffluc) enhanced green fluorescent protein (GFP) to enable detection by flow cytometry (GFP), bioluminescence-based cytotoxicity assays (ffluc), and bioluminescence imaging (ffluc) in mice. PancOVA and B16OVA cells were kindly provided by the lab of Prof. M. Huber.
Authentication	K562 used in this study were authenticated in our lab, as they<br>were directly purchased from the internationally credible vendors. PancOVA and B16OVA cell lines were authenticated by in vitro experiments.
Mycoplasma contamination	In our laboratory, the contamination of mycoplasma was regularly examined by<br>PCR. No contamination was detected while we conducted<br>experiments related to this project.
Commonly misidentified lines<br>(See ICLAC register)	No commonly misidentified cell lines were used.

Animals and other organisms

Policy information about studies involving animals; ARRIVE guidelines recommended for reporting animal research

Laboratory animals

As laboratory animals we used mice (Mus musculus).

Summary of mouse strains, mean age plus range, genetic background, sex:
CD45.1 WT (Bl6.C57 background), 10 weeks (range: 8 - 12), females
CD45.2 WT (Bl6.C57 background), 10 weeks (range: 8 - 12), females
CD45.1 OT-I (Bl6.C57 background), 10 weeks (range: 8 - 12), females
CD45.2 Ffar2−/−Ffar3−/−mice (on a C57BL/6 background), 10 weeks (range: 8 - 12), females
CD45.2 FIR×tiger (Bl6.C57 background), 10 weeks (range: 8 - 12), females
Rag1-/- (Bl6.C57 background), 10 weeks (range: 8 - 12), females

General mouse work, such as daily animal care, breeding and offspring separation, was carried out under SPF or GF conditions at the

	Biomedical Research Center, Philipps-University of Marburg. Mice are kept at 21-23°C and 40% humidity with a 12h light/ 12h dark light cycle. The sterile conditions for GF animals were routinely tested (twice a week) by culturing feces in thioglycollate medium under aerobic and anaerobic conditions for at least two weeks.
Wild animals	No wild animals were used in this study.
Field-collected samples	This study did not involve collected samples from the field
Ethics oversight	The study was approved by Regierungspräsidium Gießen, Germany (Nr. G24/2019) and conducted according to the German animal protection law.

Note that full information on the approval of the study protocol must also be provided in the manuscript.

Human research participants

Policy information about studies involving human research participants

Population characteristics	Healthy donors (m/f of various ages) from Würzburg, Germany.
Recruitment	No potential self-selection bias is known. Peripheral blood mononuclear cells (PBMCs) were isolated from healthy donors assigned to the lab by the transfusion medicine department at the University Hospital.
Ethics oversight	All participants provided written informed consent to participate in research protocols approved by the Institutional Review Board of the University of Würzburg (146/17-me).

Note that full information on the approval of the study protocol must also be provided in the manuscript.

Flow Cytometry

Plots

Confirm that:

- [x] The axis labels state the marker and fluorochrome used (e.g. CD4-FITC).
- [x] The axis scales are clearly visible. Include numbers along axes only for bottom left plot of group (a 'group' is an analysis of identical markers).
- [x] All plots are contour plots with outliers or pseudocolor plots.
- [x] A numerical value for number of cells or percentage (with statistics) is provided.

Methodology

Sample preparation	For the isolation of lymphocytes from murine spleen and LN, a single cell suspension was generated by mashing the organ through a 30 µM cell strainer. T cells were obtained by magnetic-bead seperation.<br>For the analysis of tumor-infiltrating lymphocytes, the tumor tissue was homogenized by using the Miltenyi GentleMACS protocol. T cells were obtained by magnetic-bead seperation.<br>Human PBMCs were isolated from peripheral blood of healthy donors by discontinuous percoll-gradient centrifugation. Human T cells from the PBMC fraction were obtained by magnetic-bead seperation.<br>Cells were washed in PBS before labeling with fluorochrome-conjugated<br>antibodies. Labeling of single-cell suspensions was performed on ice in PBS supplemented with 1% FCS. Cells were first incubated<br>for 10 min in PBS supplemented with 1% FCS. Labeling was performed for 10 min on ice in the dark.<br>Intracellular cytokine or transcription factor stainings were carried out upon fixation with 2% formaldehyde solution or the Transcription factor Staining Set (eBioscience) and permeabilization with saponin buffer (0.3% saponin, 2% FCS in 1x PBS).
Instrument	Analysis of flow cytometry experiments was performed on a FACSCalibur and sorting was performed on a FACS Aria III (BD Biosciences).
Software	BD FACSDiva software and BD Cellquest Pro software were used for data collection and FlowJo version 10 for analysis of data.
Cell population abundance	The purity of flow- and MACS-sorted cells was higher than 95% and were determined by flow cytometry after sorting.
Gating strategy	Based on cell size (forward scatter, FSC) and cell granularity (sideward scatter, SSC), cell debris were excluded from cells. Subsequently, FCS and SSC doublets were excluded. Cell debris, cell doublets and dead cell exclusions were applied in all flow cytometry analysis.

- [x] Tick this box to confirm that a figure exemplifying the gating strategy is provided in the Supplementary Information.
